# Orthodontic treatment need assessment and treatment timing – a questionnaire survey among specialists in Denmark and Sweden

**DOI:** 10.2340/aos.v85.46501

**Published:** 2026-07-22

**Authors:** Asta Nyvang Rank, Mikael Sonesson, Sofia Petrén, Liselotte Paulsson, Liselotte Sonnesen

**Affiliations:** a Orthodontics, Department of Odontology, Faculty of Health and Medical Sciences, University of Copenhagen, Copenhagen, Denmark; b Department of Orthodontics, Section 4, Faculty of Odontology, Malmö University, Malmö, Sweden

**Keywords:** Orthodontic treatment need, orthodontic treatment indication, orthodontic indices, clinical consensus, publicly funded treatment, malocclusions

## Abstract

**Objective:**

To analyze national and regional differences in assessment of orthodontic treatment needs and timing for treatment between specialists in orthodontics in Denmark and Sweden. Additionally, to map the use of digital platforms in orthodontic referral processes.

**Materials and methods:**

A questionnaire comprising 20 items was developed to assess nine patient cases with different malocclusions and sent to nearly 500 board-certified orthodontic specialists in Denmark and Sweden. Each case was documented by clinical photos, line drawings of the head and face, and malocclusion descriptions. Before the start of the survey, the questionnaire was tested in both countries by eight experienced orthodontists. The survey was distributed between 06 November 2024 and 27 January 2025. Data were analyzed using chi-square tests with false discovery rate correction.

**Results:**

Two hundred and nineteen responses were included. Both intra-regional and cross-national differences were found in treatment need assessments. Danish respondents showed greater agreement, while Swedish assessments varied more. Digital referral systems were more common in Sweden but linked to an increased tendency to select patient observation rather than making a definitive assessment of treatment need. In Denmark, high agreement (> 80%) was found for treatment need in six of nine cases. One patient case was generally deemed not eligible, while one case demonstrated high variability. Significant regional differences were noted in both treatment need and timing. In Sweden, strong agreement was observed for five cases. One case showed wide variation. Regional differences were significant for some cases, both in need and preferred treatment timing.

**Conclusions:**

Specialists in Orthodontics in Denmark showed more consistent assessments than those in Sweden, likely due to national guidelines. Digital referrals were common in Sweden but linked to more varied evaluations, especially in borderline cases.

## Introduction

Malocclusions, defined as deviations in occlusion, dentition, and arch space [1] represent one of the most common conditions affecting craniofacial morphology and oral function, and are ranked third in dental morbidity after periodontitis and caries [2]. The prevalence of malocclusions exhibits substantial geographic variation, influenced by both genetic predispositions and environmental determinants [3, 4]. Distal molar occlusion is particularly common in European populations, with prevalence rates reaching up to 26% among Caucasian cohorts [3, 5, 6]. However, reported prevalence rates range widely – from 39 to 93%, reflecting methodological inconsistencies and divergent referral practices across studies [3, 6, 7].

In Denmark, eligibility for publicly funded orthodontic treatment is governed by referral criteria established by the Danish Health Authority, which estimates that approximately 25% of each birth cohort presents with an objective treatment need [8–10]. However, the proportion of patients offered publicly funded orthodontic treatment varies, as all individuals who meet the established criteria are entitled to treatment. These criteria are grounded in a biological risk assessment framework, emphasizing both physical injury and psychosocial burden [8–11]. Nevertheless, referral practices may vary depending on the individual clinician’s interpretation of the guidelines, potentially leading to inequities in access to care [8, 12].

In contrast, Sweden utilizes a variety of orthodontic indices, as there is no legal requirement to follow a specific system [13, 14]. These include the Index of Orthodontic Treatment Need (IOTN) [15–19], the Index of Complexity, Outcome and Need (ICON) [20], regional indices, and the Swedish Medical Board Index (SMBI) [21, 22]. Both countries offer publicly funded orthodontic care to children and adolescents who meet the criteria, up to and including the age of 19 in Sweden and 21 in Denmark [21–24].

Referral is initiated by the child’s general dentist and performed in consultation with an orthodontist [10]. An accurate classification of malocclusions according to an index of orthodontic treatment need is critical to ensuring timely intervention [8, 11, 25]. Delayed referral may compromise treatment outcomes and increase therapeutic complexity [8, 26–29]. In addition, large deviations in the selection of patients for publicly funded orthodontic treatment may result in inequities in orthodontic treatments of children. Thus, it is pertinent to investigate whether variation exists in the assessment of treatment need – both within each country and in cross-national comparisons between Denmark and Sweden. Such an inquiry may facilitate more equitable and timelier patient selection, with potential implications for clinical efficacy and health system efficiency.

To the best of our knowledge, no previous studies have systematically evaluated whether differences exist in the assessment of treatment need within or between Denmark and Sweden. We hypothesize that such variations are present in both countries regarding the evaluation of objective treatment need and optimal treatment timing. We further hypothesize that digital technologies are more frequently employed in referral procedures in peripheral regions than in major urban areas, and that a clearer specification of existing guidelines would promote greater uniformity in access to publicly funded orthodontic care. The aims of the present study were therefore to identify national and regional differences in the evaluation of treatment need, preferred timing of treatment, and treatment strategies, as well as to map the use of digital technologies in orthodontic referral processes through national surveys in Denmark and Sweden.

## Materials and methods

The questionnaire was distributed to a total of approximately 200 board-certified orthodontic specialists in Denmark, except for Greenland and the Faroe Islands, and close to 300 board-certified orthodontic specialists in Sweden via their respective national associations: the Association of Orthodontic Specialists (FSO) in Denmark and the Swedish Orthodontic Society (SOF) in Sweden. Participants received the questionnaire with both a direct link and a QR code, ensuring convenient and straightforward access for completion.

Inclusion criteria were limited to fully completed questionnaires submitted by actively practicing orthodontic specialists working in either the public or private sector. Responses were excluded if they were left incomplete or originated from individuals not meeting the professional criteria. Specifically, submissions from retired orthodontists or dental professionals currently enrolled in orthodontic specialist training programs were excluded from the final analysis.

The study was approved by the Danish Data Protection Agency (Reference No.: 514-1038/24-3000) and the Ethics Committee at Malmö University, Sweden (Dnr. MAU 2025/2692). As all personal data were fully anonymized, formal approval was not required.

### Pilot testing

The questionnaire was subjected to pilot testing in both Denmark and Sweden. In Denmark, the pilot study encompassed five orthodontic specialists representing different employment conditions, geographic practice locations, gender identities, and levels of clinical experience. In Sweden, the pilot cohort comprised two female and one male specialists in orthodontics, each with over a decade of professional experience in orthodontic referral assessment.

The pilot data were subjected to thorough analysis, which prompted revisions to the phrasing of specific items, a restructuring of the questionnaire format, and the inclusion of a patient case exemplifying a borderline referral case.

### The questionnaire

The survey was digital and based on nine authentic patient cases reflecting different types of malocclusions, genders, and ages (Table 1). The questionnaire consisted of 20 items, four of which were repeated to specifically address the nine selected cases, and 16 focusing on more general aspects. The cases were carefully selected in consensus by the authors LS (Liselotte Sonnesen), MS (Mikael Sonesson), SP (Sofia Petrén), and LP (Liselotte Paulsson) with over 20 years of clinical and research experience in orthodontics. Each case included standard referral information: profile and frontal extraoral clinical photographs (line drawings created with an Apple pen generation 1 in Microsoft PowerPoint on an Apple iPadOS17.5.1), five intraoral clinical photographs for each case, and written descriptions of the malocclusion and functional aspects. The questionnaire distributed to Danish orthodontic specialists was developed by using University of Copenhagen survey software (SurveyXact©), while an identical version sent to the Swedish orthodontic specialists was developed by using Malmö University survey software (Artologik©). The questionnaires were available for responses from 06 November 2024 to 27 January 2025, with three reminders sent at three, six and approximately 10 weeks after initial distribution.

**Table 1:** Characteristics of the selected patient case studies that served as the foundation for the questionnaire-based investigation.

**Case 1**	9:8-year-old female patient in mixed dentition with a right-sided posterior crossbite and functional mandibular deviation, resulting in midline shift and mild facial asymmetry.	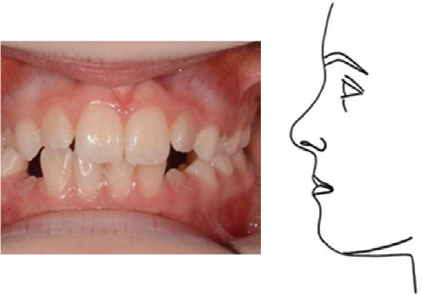
**Case 2**	8:6-year-old boy with mandibular overbite, unilateral posterior crossbite on the right side, and a retruded edge-to-edge incisal position.	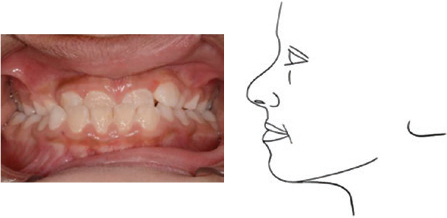
**Case 3**	13:2-year-old girl with few interproximal tooth contacts and limited occlusal stability.	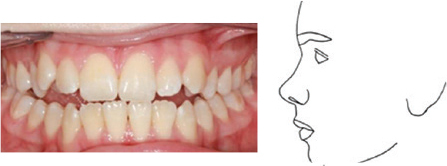
**Case 4**	10:1-year-old girl with fully developed dentition, with palatal impingement, and no ectopic eruption in the canine region (3+3).	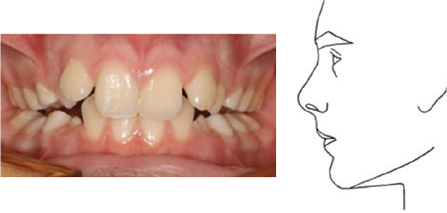
**Case 5**	13:5-year-old boy with fully developed dentition, 12 mm maxillary overjet, Class I molar relationship, and insufficient lip seal.	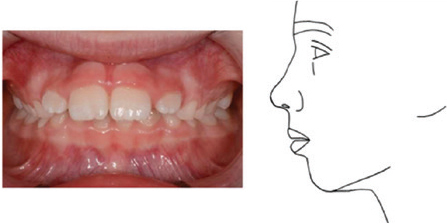
**Case 6**	8:2-year-old girl in mixed dentition, 11 mm maxillary overjet, Class I molar relationship, and insufficient lip seal.	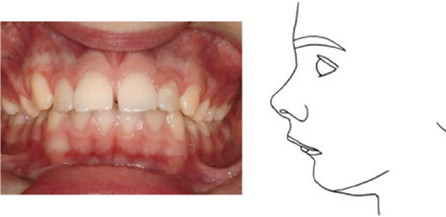
**Case 7**	10:3-year-old girl in mixed dentition, bilateral posterior crossbite with no functional mandibular deviation.	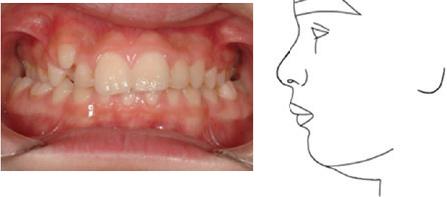
**Case 8**	14:2-year-old girl with fully developed dentition, crowding in both jaws resulting in maxillary high labially canines	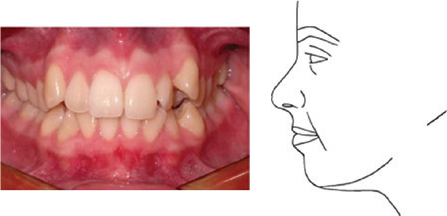
**Case 9**	14:6-year-old girl with fully developed dentition, crowding within both jaws, more severe in the lower jaw.	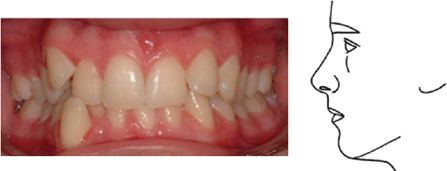

### Statistical analyses

Descriptive statistics are presented in frequency in Table 2. In addition, χ² tests were performed on explanatory variables related to the assessment of eligibility for publicly funded treatment, preferred timing for treatment initiation, and tentative treatment plans. χ² tests were applied to evaluate potential statistically significant differences between responses to questions 17.1–9 (Is there an indication for publicly funded orthodontic treatment?) and 19.1–9 (If the patient is offered treatment, when would you prefer to initiate it?) across the two countries.

**Table 2:** Descriptive overview of Danish and Swedish participant characteristics.

Characteristics	Category	Danish respondents	Swedish respondents
		% (*n*)	% (*n*)
Gender	Female	69.4 (75)	62.2 (69)
	Male	30.6 (33)	37.8 (42)
	Other	0 (0)	0 (0)
Age	25–30 years	0 (0)	0 (0)
	31–40 years	14.8 (16)	14.4 (16)
	41–50 years	36.1 (39)	36.0 (40)
	51–60 years	19.4 (21)	25.2 (28)
	61–70 years	22.2 (24)	13.5 (15)
	> 70 years	7.4 (8)	10.8 (12)
Years in general dental practice before orthodontic specialization	2–4 years	42.6 (46)	19.8 (22)
	5–7 years	35.2 (38)	35.1 (39)
	8–10 years	12.0 (13)	25.2 (28)
	>11 years	10.2 (11)	19.8 (22)
Years as a certified orthodontic specialist	0–2 years	4.6 (5)	10.8 (12)
	3–6 years	13.9 (15)	16.2 (18)
	7–10 years	16.7 (18)	9.9 (11)
	11–15 years	19.4 (21)	15.3 (17)
	16–20 years	15.7 (17)	24.3 (27)
	> 20 years	29.6 (32)	23.4 (26)
Number of workplaces employed at	One workplace	56.6 (61)	82.9 (92)
	Multiple workplaces	43.5 (47)	17.1 (19)
Total		100 (108)	100 (111)

To correct for multiple testing, False Discovery Rate (FDR) was estimated using the Benjamini-Hochberg method. Statistical analyses were conducted using IBM Statistical Package for the Social Sciences (IBM SPSS), Version 29.0.2.0 for Windows. Results were considered statistically significant at *p*-values < 0.05.

## Results

The dataset comprised a total of 219 responses, of which 108 originated from the questionnaire distributed in Denmark and 111 from its counterpart distributed in Sweden. According to official records from the Danish Health Authority (Sundhedsstyrelsen), a total of 163 specialist orthodontists were engaged in active clinical practice in Denmark in 2023 [30], yielding an overall response rate of 66.2% for the present investigation. Furthermore, data published by the Swedish National Board of Health and Welfare (Socialstyrelsen) indicate that 312 specialist orthodontists were practicing in Sweden in 2024 [31]. Owing to the absence of reliable information regarding the proportion of actively practicing specialists within the membership of SOF, it was not feasible to accurately ascertain the representativeness of the Swedish sample. Under the hypothetical assumption that all practicing Swedish specialist orthodontists were affiliated with SOF, the estimated response rate would amount to 35.6%. Descriptive statistical results of the background data are provided for Denmark and Sweden in Table 2.

No associations remained statistically significant after adjustment for multiple testing using FDR, indicating the absence of robust effects under the applied significance threshold (Table 3). Overall, the results revealed the presence of both intra-regional and cross-national variations in the assessment of treatment need (Tables 4–6). In Denmark, where the national referral criteria issued by the Danish Health Authority are legally mandated, respondents showed greater agreement than in Sweden, where different orthodontic indices are used regionally (Table 7). Digital referral systems were significantly more prevalent in Sweden; however, their use was associated with the respondent being more likely to recommend observation of the patient, specifically regarding patient case 4. PEXIP (formerly My Meeting Video) and Microsoft Teams are digital platforms utilized to facilitate remote consultations.

**Table 3:** *P*-values from chi-squared and FDR-corrected *p*-values.

Characteristic	Patient case	Sweden		Denmark	
		V.17.1-9*	V19.1-9**	V.17.1-9*	V19.1-9**
		*P*-value (FDR-corrected)	*P*-value (FDR-corrected)	*P*-value (FDR-corrected)	*P*-value (FDR-corrected)
Gender	1	0.324 (0.729)	0.057 (0.057)	0.168 (0.430)	0.315 (0.490)
	2	0.433 (0.779)	0.932 (0.932)	-	0.168 (0.378)
	3	0.165 (0.729)	0.066 (0.066)	0.4 (0.533)	0.443 (0.569)
	4	0.064 (0.576)	0.106 (0.106)	0.281 (0.449)	0.103 (0.378)
	5	0.324 (0.729)	0.217 (0.217)	0.166 (0.430)	0.033 (0.297)
	6	0.87 (0.950)	0.772 (0.772)	0.215 (0.430)	0.327 (0.667)
	7	0.95 (0.950)	0.686 (0.686)	0.933 (0.933)	0.951 (0.490)
	8	0.863 (0.950)	0.019 (0.650)	0.168 (0.430)	0.107 (0.951)
	9	0.815 (0.950)	0.323 (0.323)	0.474 (0.541)	0.145 (0.378)
Age	1	0.064 (0.252)	0.13 (0.130)	0.303 (0.654)	0.061 (0.133)
	2	0.559 (0.718)	0.636 (0.636)	-	0.103 (0.154)
	3	0.084 (0.252)	0.204 (0.204)	0.763 (0.848)	0.074 (0.133)
	4	0.471 (0.706)	0.653 (0.653)	0.822 (0.848)	0.237 (0.304)
	5	0.643 (0.723)	0.386 (0.386)	0.336 (0.654)	0.033 (0.133)
	6	0.238 (0.428)	0.014 (0.014)	0.848 (0.848)	0.073 (0.133)
	7	0.037 (0.252)	0.489 (0.489)	0.147 (0.654)	0.746 (0.746)
	8	0.229 (0.428)	0.445 (0.445)	0.217 (0.654)	0.288 (0.324)
	9	0.864 (0.864)	0.648 (0.648)	0.409 (0.654)	0.064 (0.133)
Years in general dental practice before orthodontic specialization	1	0.431 (0.682)	0.665 (0.665)	0.673 (0.782)	0.548 (0.756)
	2	0.393 (0.682)	0.36 (0.360)	-	0.673 (0.756)
	3	0.674 (0.866)	0.769 (0.769)	0.41 (0.782)	0.689 (0.756)
	4	0.944 (0.944)	0.435 (0.435)	0.969 (0.969)	0.273 (0.756)
	5	0.228 (0.682)	0.566 (0.566)	0.572 (0.782)	0.155 (0.697)
	6	0.158 (0.682)	0.161 (0.161)	0.541 (0.782)	0.756 (0.756)
	7	0.455 (0.682)	0.847 (0.847)	0.685 (0.782)	0.021 (0.189)
	8	0.027 (0.243)	0.028 (0.028)	0.221 (0.782)	0.362 (0.756)
	9	0.848 (0.944)	0.645 (0.645)	0.571 (0.782)	0.751 (0.756)
Years as a certified orthodontic specialist	1	0.757 (0.786)	0.832 (0.832)	0.59 (0.786)	0.514 (0.578)
	2	0.349 (0.533)	0.36 (0.360)	-	0.197 (0.421)
	3	0.264 (0.533)	0.135 (0.135)	0.46 (0.786)	0.062 (0.279)
	4	0.233 (0.533)	0.463 (0.463)	0.541 (0.786)	0.416 (0.534)
	5	0.786 (0.786)	0.612 (0.612)	0.799 (0.832)	0.234 (0.421)
	6	0.415 (0.533)	0.282 (0.282)	0.019 (0.152)	0.187 (0.421)
	7	0.091 (0.533)	0.436 (0.436)	0.05 (0.200)	0.591 (0.591)
	8	0.384 (0.533)	0.461 (0.461)	0.563 (0.786)	0.362 (0.534)
	9	0.383 (0.533)	0.604 (0.604)	0.832 (0.832)	0.788 (0.000)
Number of workplaces employed at	1	0.079 (0.118)	0.031 (0.031)	0.045 (0.212)	0.365 (0.529)
	2	0.027 (0.081)	0.166 (0.166)	-	0.412 (0.529)
	3	0.022 (0.081)	0.006 (0.006)	0.14 (0.362)	0.381 (0.529)
	4	0.071 (0.118)	0.423 (0.423)	0.215 (0.362)	0.493 (0.554)
	5	0.079 (0.118)	0.154 (0.154)	0.265 (0.362)	0.185 (0.529)
	6	0.021 (0.081)	0.11 (0.110)	0.272 (0.362)	0.41 (0.529)
	7	0.632 (0.676)	0.076 (0.076)	0.053 (0.212)	0.296 (0.529)
	8	0.676 (0.676)	0.294 (0.294)	0.602 (0.602)	0.051 (0.459)
	9	0.252 (0.324)	0.48 (0.480)	0.477 (0.545)	0.589 (0.589)
Treatment waiting time	1	0.799 (0.865)	0.89 (0.890)	0.14 (0.576)	0.805 (0.857)
	2	0.638 (0.865)	0.01 (0.010)	-	0.857 (0.857)
	3	0.865 (0.865)	0.432 (0.432)	0.924 (0.924)	0.597 (0.857)
	4	0.285 (0.711)	0.589 (0.589)	0.766 (0.924)	0.773 (0.857)
	5	0.799 (0.865)	0.371 (0.371)	0.158 (0.576)	0.33 (0.742)
	6	0.026 (0.117)	0.118 (0.118)	0.81 (0.924)	0.071 (0.639)
	7	0.008 (0.072)	0.073 (0.073)	0.216 (0.576)	0.442 (0.795)
	8	0.57 (0.865)	0.379 (0.379)	0.511 (0.924)	0.189 (0.742)
	9	0.316 (0.711)	0.632 (0.632)	0.672 (0.924)	0.274 (0.742)

The tests were between dependent variables and, respectively, the assessment of treatment need, the optimal timing for treatment initiation, and the tentative treatment. FDR: false discovery rate.

V17.1–9 refers to question 17 in the questionnaire for each of the nine patient case studies. The question was as follows: 17.1–9. Is there an indication for publicly funded orthodontic treatment?

V19.1–9 refers to question 19 in the questionnaire for each of the nine patient case studies. The question was as follows: 19.1–9. If the patient is offered treatment, when would you prefer to initiate it?

**Table 4:** Regional breakdown by Danish geographical areas of the assessment of treatment need for the nine patient cases.

17.1-9. Is there an indication for publicly funded orthodontic treatment?							
Patient case	Capital City Region % (*n*)	Region Zealand % (*n*)	North Jutland Region % (*n*)	Mid Jutland Region % (*n*)	Region of Sourthern Denmark % (*n*)	Multiple regions % (*n*)	Other % (*n*)
**Patientcase 1**							
Yes	100 (45)	78.6 (11)	100 (8)	100 (16)	100 (11)	100 (12)	100 (2)
No	0.0 (0)	21.4 (3)	0.0 (0)	0.0 (0)	0.0 (0)	0.0 (0)	0.0 (0)
Observation	0.0 (0)	0.0 (0)	0.0 (0)	0.0 (0)	0.0 (0)	0.0 (0)	0.0 (0)
**Patientcase 2**							
Yes	100 (45)	100 (14)	100 (8)	100 (16)	100 (11)	100 (12)	100 (2)
No	0.0 (0)	0.0 (0)	0.0 (0)	0.0 (0)	0.0 (0)	0.0 (0)	0.0 (0)
Observation	0.0 (0)	0.0 (0)	0.0 (0)	0.0 (0)	0.0 (0)	0.0 (0)	0.0 (0)
**Patientcase 3**							
Yes	91.1 (41)	100 (14)	50 (4)	87.5 (14)	72.7 (8)	66.7 (8)	50 (1)
No	0.0 (0)	0.0 (0)	0.0 (0)	0.0 (0)	0.0 (0)	0.0 (0)	0.0 (0)
Observation	8.9 (4)	0.0 (0)	50 (4)	12.5 (2)	27.3 (3)	33.3 (4)	50 (1)
**Patientcase 4**							
Yes	73.3 (33)	42.9 (6)	62.5 (5)	68.7 (11)	63.6 (7)	75 (9)	50 (1)
No	2.2 (1)	0.0 (0)	0.0 (0)	6.3 (1)	0.9 (0)	0.9 (0)	0.0 (0)
Observation	24.5 (11)	57.1 (8)	37.5 (3)	25 (4)	36.4 (4)	25 (3)	50 (1)
**Patientcase 5**							
Yes	100 (45)	78.6 (11)	100 (8)	100 (16)	100 (11)	100 (12)	50 (1)
No	0.0 (0)	7.1 (1)	0.0 (0)	0.0 (0)	0.0 (0)	0.0 (0)	0.0 (0)
Observation	0.0 (0)	14.3 (2)	0.0 (0)	0.0 (0)	0.0 (0)	0.0 (0)	50 (1)
**Patientcase 6**							
Yes	95.6 (43)	92.9 (13)	100 (8)	87.5 (14)	90.9 (10)	91.7 (11)	50 (1)
No	0.0 (0)	0.0 (0)	0.0 (0)	0.0 (0)	0.0 (0)	0.0 (0)	0.0 (0)
Observation	4.4 (2)	7.1 (1)	0.0 (0)	12.5 (2)	9.1 (1)	8.3 (1)	50 (1)
**Patientcase 7**							
Yes	35.6 (16)	3.7 (4)	0.0 (0)	31.3 (5)	36.4 (4)	25 (3)	0.0 (0)
No	20 (9)	2.8 (3)	50 (4)	43.7 (7)	27.2 (3)	41.7 (5)	0.0 (0)
Observation	44.4 (20)	6.5 (7)	50 (4)	25 (4)	36.4 (4)	33.3 (4)	100 (2)
**Patientcase 8**							
Yes	4.4 (2)	0.0 (0)	12.5 (1)	6.3 (1)	0.0 (0)	0.0 (0)	0.0 (0)
No	84.5 (38)	85.7 (12)	87.5 (7)	93.7 (15)	81.8 (9)	75 (9)	50 (1)
Observation	11.1 (5)	14.3 (2)	0.0 (0)	0.0 (0)	18.2 (2)	25 (3)	50 (1)
**Patientcase 9**							
Yes	60 (27)	42.9 (6)	75 (6)	81.2 (13)	72.7 (8)	66.6 (8)	50 (1)
No	22.3 (10)	21.4 (3)	12.5 (1)	6.3 (1)	18.2 (2)	16.7 (2)	0.0 (0)
Observation	17.7 (8)	35.7 (5)	12.5 (1)	12.5 (2)	9.1 (1)	16.7 (2)	50 (1)
**Total**	100 (45)	100 (14)	100 (8)	100 (16)	100 (11)	100 (12)	100 (2)

V17.1–9 refers to question 17 in the questionnaire for each of the nine patient case studies. The question was as follows: 17.1–9. Is there an indication for publicly funded orthodontic treatment?

**Table 5:** Regional breakdown by Swedish geographical areas of the assessment of treatment need for the nine patient cases.

17.1-9. Is there an indication for publicly funded orthodontic treatment?					
Patient case	North* % (*n*)	South* % (*n*)	East* % (*n*)	West* % (*n*)	Total % (*n*)
**Patientcase 1**					
Yes	100 (17)	100 (21)	97.7 (42)	96.6 (28)	98.2 (108)
No	0.0 (0)	0.0 (0)	0.0 (0)	3.4 (1)	0.9 (1)
Observation	0.0 (0)	0.0 (0)	2.3 (1)	0.0 (0)	0.9 (1)
**Patientcase 2**					
Yes	100 (17)	100 (21)	97.7 (42)	100 (29)	99.1 (109)
No	0.0 (0)	0.0 (0)	0.0 (0)	0.0 (0)	0.0 (0)
Observation	0.0 (0)	0.0 (0)	2.3 (1)	0.0 (0)	0.9 (1)
**Patientcase 3**					
Yes	41.2 (7)	85.7 (18)	37.2 (16)	51.7 (15)	50.9 (56)
No	0.0 (0)	0.0 (0)	23.3 (10)	17.2 (5)	13.6 (15)
Observation	58.8 (10)	14.3 (3)	39.5 (17)	31.1 (9)	35.5 (39)
**Patientcase 4**					
Yes	82.4 (14)	66.7 (14)	41.9 (18)	48.3 (14)	54.5 (60)
No	0.0 (0)	0.0 (0)	11.6 (5)	17.2 (5)	9.1 (10)
Observation	17.6 (3)	33.3 (7)	46.5 (20)	34.5 (10)	36.4 (40)
**Patientcase 5**					
Yes	94.1 (16)	100 (21)	100 (43)	96.6 (28)	98.2 (108)
No	0.0 (0)	0.0 (0)	0.0 (0)	3.4 (1)	0.9 (1)
Observation	5.9 (1)	0.0 (0)	0.0 (0)	0.0 (0)	0.9 (1)
**Patientcase 6**					
Yes	100 (17)	100 (21)	93 (40)	100 (29)	97.3 (107)
No	0.0 (0)	0.0 (0)	0.0 (0)	0.0 (0)	0.0 (0)
Observation	0.0 (0)	0.0 (0)	7 (3)	0.0 (0)	2.7 (3)
**Patientcase 7**					
Yes	29.4 (5)	19 (4)	35.6 (11)	51.7 (15)	31.8 (35)
No	11.8 (2)	28.6 (6)	30.2 (13)	27.6 (8)	28.1 (31)
Observation	58.8 (10)	52.4 (11)	44.2 (19)	20.7 (6)	40.1 (45)
**Patientcase 8**					
Yes	35.3 (6)	80.9 (17)	65.1 (28)	51.7 (15)	60 (66)
No	53.9 (9)	14.3 (3)	32.6 (14)	44.9 (13)	35.5 (39)
Observation	11.8 (2)	4.8 (1)	2.3 (1)	3.4 (1)	4.5 (5)
**Patientcase 9**					
Yes	94.1 (16)	90.5 (19)	95.3 (41)	96.6 (28)	94.5 (104)
No	5.9 (1)	9.5 (2)	4.7 (2)	3.4 (1)	5.5 (6)
Observation	0.0 (0)	0.0 (0)	0.0 (0)	0.0 (0)	0.0 (0)
**Total**	100 (17)	100 (21)	100 (43)	100 (29)	100 (110)

V17.1–9 refers to question 17 in the questionnaire for each of the nine patient case studies. The question was as follows: 17.1–9. Is there an indication for publicly funded orthodontic treatment?

* **North:** Jämtland, Härjedalen, Norrbotten, Västerbotten, Västernorrland, **South:** Blekinge, Kalmar, Kronoberg, Skåne, Södra Halland, **East:** Dalarna, Gotland, Gävleborg, Uppland, Stockholm, Södermanland, Sörmland, Västmanland, Örebro län, Östragötaland, **West:** Jönköping, Norra Halland, Värmland, Västra Götaland, (Kungsbacka, Varbeg, Falkenberg).

**Table 6:** Chi-square test of the assessment of treatment need in relation to Danish and Swedish regions.

Patient case	Denmark		Sweden	
	*x*²	*P*	*x*²	*P*
Patient case 1	20.718	0.002	4.373	0.626
Patient case 2	-	-	1.572	0.666
Patient case 3	16.251	0.12	20.913	0.002
Patient case 4	8.723	0.726	13.268	0.039
Patient case 5	33.033	< 0.001	8.322	0.215
Patient case 6	7.173	0.305	4.805	0.187
Patient case 7	12.451	0.410	11.808	0.066
Patient case 8	11.450	0.491	11.220	0.082
Patient case 9	8.847	0.716	0.960	0.811

**Table 7:** Agreement in the assessment of tentative treatment among respondents in Denmark and Sweden.

Level of agreement	Danish orthodontic specialists	Swedish orthodontic specialists
Patient cases with clear treatment indication (> 80%)	Patient case 1, 2, 3, 5 and 6	Patient case 1, 2, 5, 6 and 9
Patient cases without treatment indication (> 80%)	Patient case 8	-
Patient cases with moderate agreement on treatment indication (> 50; < 80)	Patient case 4 and 9	Patient case 3, 4 and 8
Patient cases with wide variation in assessment of treatment indication (< 50)	Patient case 7	Patient case 7

### Data from the questionnaire distributed in Denmark

For patient case 1 (97.2%; *n* = 105), case 2 (100%; *n* = 108), case 3 (83.3%; *n* = 90), case 5 (96.3%; *n* = 104), and case 6 (92.6%; *n* = 100), a high level of agreement was observed among respondents from Denmark regarding the presence of a treatment need in accordance with the national referral criteria (> 80% agreement). Additionally, there was strong agreement that patient case 8 did not warrant publicly funded orthodontic treatment (Table 7). Patient case 7 exhibited the lowest agreement in responses, with the majority opting for observation rather than making a definitive judgment based on the available material.

For the remaining patient cases, there was less agreement regarding the assessment of treatment need; however, a slight majority of the Danish respondents still believed that treatment was warranted.

Statistically significant regional differences were observed in the assessment of treatment need for patient case 1, where Region Zealand demonstrated a marked difference as the only region with respondents who did not identify a treatment need (*p* = 0.002). For patient case 3, Region Zealand was the only region to reach unanimous agreement (*p* = 0.012), and for patient case 5, it was also the only region displaying inconsistent evaluations (*p* < 0.001).

Statistically significant regional differences were observed in the preferred timing for treatment initiation across several patient cases. For patient case 1, respondents from Region Zealand and Region of Southern Denmark differed from other regions by indicating a preference for treatment initiation during the pubertal growth spurt (*p* = 0.005), whereas respondents from the remaining regions preferred initiation before the pubertal growth spurt. Regarding patient case 2, greater variation in responses was noted in Capital City Region and Region Zealand compared to the other regions (*p* = 0.004). Regarding patient case 4, Capital City Region showed a marked spread in assessments regarding treatment timing (*p* < 0.001). For patient case 5, Region of Southern Denmark was the only region where respondents reached full agreement on the timing of treatment (*p* < 0.001).

### Data from the questionnaire distributed in Sweden

For patient case 1 (98.2%; *n* = 109), case 2 (99.1%; *n* = 110), case 5 (98.2%; *n* = 109), case 6 (97.3%; *n* = 108), and case 9 (94.6%; *n* = 105), the Swedish respondents agreed in their assessment of treatment need, specifically that treatment was indicated (> 80% agreement). For patient case 7, the same was observed among the Swedish respondents as among the Danish, with a wide variation in the assessment of treatment need. For the remaining patient cases, there was less agreement regarding the assessment of treatment need; however, a slight majority of respondents still believed that treatment was warranted.

Statistically significant differences were found in the assessment of orthodontic treatment need between regions of Sweden for patient case 3 (*p* < 0.05) and patient case 4 (*p* < 0.05). The northern and southern parts of Sweden demonstrated the most consistent evaluations regarding treatment need (Tables 5 and 6). A statistically significant difference was also observed between regions in terms of when specialists prefer to initiate orthodontic treatment for patient case 3 (*p* = 0.007) and case 4 (*p* = 0.017).

### The use of digital platforms and infrastructure in orthodontic consultation processes

Analyses on the Danish data regarding digital consultations and agreements concerning treatment need were not performed, as only one respondent from Region Southern Denmark indicated that digital consultation was used. Among the Swedish respondents, who indicated that digital referral was possible, the most common method was the use of photographic or study model documentation sent to the referring orthodontist (47.7%, *n* = 42). Digital referrals via video consultation with the patient at the general dental clinic were less frequently used (15.9%, *n* = 14), and the rest of the respondents reported using other referral methods (36.4%, *n* = 32). Among the Swedish respondents, a significant regional difference was observed in the use of digital consultations (*p* < 0.001). In the northern part of Sweden, all respondents (100%; *n* = 17) reported that digital referral was possible, which distinguished this region from the other Swedish regions (Table 8). In patient case 4, a statistically significant difference in treatment need was observed depending on whether digital consultation was possible or not (*p* = 0.041). Specifically for this case, respondents who indicated that digital referrals were possible were more likely to recommend observation of the patient, compared to those who relied solely on the physical referral.

**Table 8:** Use of digital platforms and infrastructure in orthodontic consultation processes in relation to Swedish regions.

Region	Yes	No	Total
	% (*n*)	% (*n*)	% (*n*)
North	15.5 (17)	0.0 (0)	15.5 (17)
South	7.3 (8)	11.8 (13)	20.0 (22)
East	36.4 (40)	2.7 (3)	39.1 (43)
West	20.9 (23)	5.45 (6)	26.4 (29)
Total	80 (88)	20.0 (22)	100 (110)

**North:** Jämtland, Härjedalen, Norrbotten, Västerbotten, Västernorrland, **South:** Blekinge, Kalmar, Kronoberg, Skåne, Södra Halland, **East:** Dalarna, Gotland, Gävleborg, Uppland, Stockholm, Södermanland, Sörmland, Västmanland, Örebro län, Östragötaland, **West:** Jönköping, Norra Halland, Värmland, Västra Götaland, (Kungsbacka, Varbeg, Falkenberg).

## Discussion

This study provides novel and important insights into the variability in orthodontic treatment need assessments among specialists in orthodontics in Denmark and Sweden. The findings reveal both regional and cross-national differences in the evaluation of treatment need, timing of intervention, and choice of therapeutic strategy. These discrepancies reflect not only divergent clinical practices but also structural and organizational differences within the healthcare systems of the two countries.

In Denmark, the use of legally mandated national referral criteria issued by the Danish Health Authority [8–11] has resulted in a higher degree of agreement among orthodontic specialists regarding treatment need. In contrast, Sweden employs various orthodontic indices – such as IOTN [15–19], ICON [20], regional indices, and SMBI [21, 22]. The lack of use of one national standardized index in Sweden appears to contribute to greater variability in clinical assessments, consistent with previous research highlighting interpretive challenges associated with subjective indices [32].

Statistically significant regional differences were identified in the assessment of specific patient cases in both Denmark and Sweden. Notably, cases involving bilateral crossbite without functional disturbances and deep bite with palatal impingement exhibited substantial variation in perceived treatment need. These conditions fall within a treatment need grey zone where existing indices fail to offer sufficient differentiation. ICON is highlighted as a potentially valuable tool for quantifying the severity of crowding and thereby supporting more objective clinical judgments [20].

The findings highlight regional differences in clinical judgment and decision-making, which may have implications for consistency in referral practices and treatment planning across the country. The observed variation in clinical judgments – both within and across national borders – emphasizes the need for more precise and standardized referral criteria. A clearer definition of treatment need could help reduce inequities in access to care and enhance the cost-effectiveness of orthodontic services [33]. Moreover, standardized criteria may improve patient communication, equity in publicly funded orthodontic treatment and reduce the incidence of complaints [34].

Digital orthodontic consultations were significantly more prevalent in Sweden than in Denmark, likely reflecting differences in organizational infrastructure and technological implementation [35]. However, the results indicated that digital consultations were associated with greater variability in clinical assessments, suggesting that such systems may not fully substitute for in-person evaluations. This finding aligns with prior studies emphasizing the need for supplementary clinical examination within digital referral workflows [35].

However, digital orthodontic consultations in Sweden encompass a range of organizational models rather than a single standardized procedure, which was also seen in the results of our investigation. Across different regions, these consultations may take the form of synchronous real-time video meetings involving the patient, the general dentist, and the orthodontist, or rely on an asynchronous system in which clinical registrations and dental records are submitted by the general dentist for later evaluation by the orthodontist. In some regions, all referrals are transmitted digitally, allowing the orthodontist to determine whether the patient should be examined in person or assessed solely based on the submitted digital material. Additionally, the model in which the orthodontist conducts in-person consultations at the public dental clinic remains in use in several parts of the country. In this study, the term ‘digital consultation’ therefore refers to this spectrum of approaches currently applied in Swedish orthodontic practice.

In the present study, clinical digital intraoral photographs and craniofacial illustrations were used for assessment of orthodontic treatment need. In previous methodological studies on the reliability and validity of intraoral photographs in assessing orthodontic treatment need [36, 37], a comparable interrater agreement in orthodontic index assessments was presented, regardless of using solely intraoral photographs or photographs and dental casts. However, there seem to be some differences in validity between the use of various indices when assessing treatment need on intraoral photographs [36]. The use of the IOTN index seems to result in high validity, which suggests that the choice of index needs to be considered when assessment of orthodontic treatment needs is made only by photographs.

The cross-sectional design and use of patient case scenarios in the present study may offer a realistic representation of clinical decision-making. However, the relatively low response rate in Sweden limits the generalizability of the findings. Moreover, Sweden is geographically substantially larger than Denmark, encompassing approximately 10 times the land area and a considerably larger population, which contributes to a higher degree of demographic, regional, and professional heterogeneity. Regional variations in both the financing and organization of orthodontic care in Sweden, as well as variations between Sweden and Denmark, certainly influence how clinical services are structured and delivered. Thus, financial factors may inadvertently influence treatment initiations, but the present study did not aim to analyze these systems as well as treatment initiation thresholds. However, differences in geographical distribution, practice settings, and access to specialist services should be considered when interpreting cross-national findings. The anonymity of responses helps mitigate the risk of social desirability bias [38], and pilot testing contributed to the validity of the questionnaire instrument [39, 40].

## Conclusion

Findings from the present study highlight differences in the assessment of orthodontic treatment needs among specialists in Denmark and Sweden – both within national boundaries and across countries. Analysis of nine patient cases revealed that certain malocclusion types – such as pronounced maxillary overjet and unilateral crossbite with functional impairment – were generally assessed consistently and clearly fell within the referral criteria in both countries. In contrast, cases involving bilateral crossbite without functional impairment, deep bite with palatal impingement, and high-labially positioned canines exhibited greater variability in evaluation, suggesting that these conditions occupy a diagnostic grey zone.

In Denmark, where the national referral criteria issued by the Danish Health Authority are legally mandated, a higher degree of agreement among orthodontic specialists was observed. In Sweden, where different orthodontic indices are applied depending on the region, as well as differences in organization of the orthodontic care, assessments were somewhat more heterogeneous.

The study further demonstrates that digital referral procedures are significantly more widespread in Sweden than in Denmark, and that the use of digital referral systems is associated with greater variability in case assessments compared to in-person evaluations.

## Data Availability

The data underlying this article will be shared upon reasonable request to the corresponding author.
